# The experiences of the families of patients admitted to the intensive care unit

**DOI:** 10.1186/s12912-024-02103-8

**Published:** 2024-06-25

**Authors:** Neda Asadi, Fatemeh Salmani

**Affiliations:** 1 Nursing Research Center, Kerman University of Medical Sciences, Kerman, Iran; 2 Nursing and Midwifery Sciences Development Research Center, Najafabad Branch, Islamic Azad University, Najafabad, Iran

**Keywords:** Family members, Hospitalization, Intensive care units

## Abstract

**Background:**

The admission of close family members to intensive care units can cause significant stress and anxiety for both patients and their families. The sudden and unexpected nature of such admissions often leaves families feeling worried, confused, and shocked. This study aimed to explore the experiences of families with loved ones admitted to the intensive care unit.

**Method:**

The current qualitative study used conventional content analysis. The researchers purposefully selected 11 close family members of patients admitted to the intensive care unit. Semi-structured in-depth face-to-face interviews were conducted with the participants. These interviews were recorded, transcribed, and analyzed the data.

**Findings:**

After reviewing and analyzing the data, three themes and nine categories emerged. These themes included the search for support resources, psychological consequences within the family, and the presence of various needs within the families.

**Conclusion:**

The study findings revealed that families, when present in the intensive care unit, actively sought support resources due to their fear of their loved one’s mortality. The interactions with the healthcare team and the fulfillment of their needs could significantly affect their sense of hope and confidence in the patient’s condition. It is recommended that nurse managers, who possess a genuine perception of the family’s needs, implement family-oriented measures and interventions to provide the necessary support.

## Introduction

Admission to the intensive care unit (ICU) can be an extremely distressing experience, leading to physical and psychological symptoms for both patients and their families [1]. The sudden and unexpected nature of ICU admission can act as a stressor, causing a crisis within the family [2]. Researchers argue that families often suffer more than patients themselves, as patients may struggle to fully comprehend the situation due to their condition [3]. Providing care not only for patients but also for their families is crucial in enhancing the quality of intensive care [4].

The primary concerns of families of ICU patients revolve around long-term hospitalization in the ICU, the possibility of death, serious complications, and the presence of complex medical devices and equipment [5]. Families who struggle to adapt to these circumstances may experience mental and emotional distress, which can disrupt family cohesion and daily life [6]. Additionally, the unique circumstances surrounding ICU patients, including therapeutic decision-making, can lead to confusion and instability within interpersonal relationships among family members [7]. A significant portion of ICU patients is unable to participate in their treatment decisions, placing additional pressure on family members who must make these decisions [8]. The financial burden resulting from hospitalization and intensive care, as well as the potential loss of employment or job instability, further exacerbates the negative experiences faced by families during a patient’s admission [9]. Systematically, families of patients experience psychological (stress, anxiety and fear, confusion and financial worries, and being under pressure from the caregiver), physical (sleep disturbances and exhaustion), social (absenteeism from work, challenges in fulfilling their roles, and lack of sufficient family support), and spiritual problems (feeling emptiness caused by the meaninglessness of life, loss of hope, lack of motivation, and damage to fundamental religious beliefs). These problems can have a significant impact on the well-being of the family. Considering that the family is the first source of support for the patient, it is necessary to address these issues through structured interviews to prevent harm and develop appropriate planning (10–11). Several studies have highlighted the experiences of families with patients hospitalized in ICUs by focusing on family planning, decision-making for the patient, and improving patient care. For instance, Leong (2023) in Malaysia emphasized the use of family experiences for systematic planning [12]. Digby (2023) in Australia highlighted the role of family experiences as intermediaries between the patient and the doctor in decision-making [13]. Alexania (2018) in Canada emphasized using family experiences to enhance patient care and develop strategies to address their problems [14]. Unfortunately, despite conducting studies in this field in Iran, there has not been a written program to address the problems faced by families with patients hospitalized in the ICU. The needs of these people, which can be identified through receiving their experiences, remain unsolved, leaving families confused and worried outside the doors of the ICU. Recognizing the importance of understanding the experiences of family members to develop effective supportive interventions healthcare professionals [15] should consider using qualitative research methods. These methods are well-suited for exploring complex issues, as critical situations and personal experiences are multidimensional and influenced by various factors within a social context. Therefore, a qualitative approach that delves into the lived experiences of individuals constantly facing these challenges is necessary to gain a comprehensive understanding. This study aimed to investigate the experiences of families with patients admitted to ICUs.

## Methods

### Design

This qualitative study was conducted using the conventional content analysis method. This method involves analyzing written, spoken, or visual messages to describe a particular phenomenon. The raw data were summarized and categorized through inferences, interpretation, and in-depth analysis. Categories and their names were extracted from the context of the data [16].

### Sample and setting

One hospital in southeastern Iran was chosen for this study. The hospital has a 75-year history and a total of 350 beds. It is divided into four ICU departments, each with 14 beds. The hospital specializes in trauma care. The patient-to-nurse ratio in the ICUs is two to one. Families of patients who had been in the ICU for at least three days were invited to participate in the study [12]. Visits in the ICU were limited to specific days, namely Monday, Wednesday, and Friday. The nurse assigned to each patient communicated with the patient’s family, providing them with necessary information. Most of the time, the patient’s family members stay behind the ward door on chairs, as there are no specific facilities available for resting or eating.

Participants were selected using a purposeful sampling from the family members of patients who had rich experiences regarding the study subject and were willing to participate in the study. The sampling process aimed to achieve maximum variation in terms of participants’ gender, educational level, and relationship to the patient. Initially, purposive sampling was employed, followed by theoretical sampling until data saturation was reached.

One of the researchers was responsible for the purposeful sampling. In this study, theoretical sampling was used based on the concepts obtained from data analysis. This approach aimed to refine and establish proper communication between categories rather than creating new ones. The selection of participants for interviews was based on the inclusion criteria, focusing on individuals who showed better interaction and had more involvement with patient hospitalization. Before each interview, participants were provided with a clear explanation of the study’s purpose, approximate duration, and assurance of anonymity. They were given enough time to read the informed consent form and make an informed decision about whether to participate in the interview.

### Data collection

The study was conducted from July to October 2022. Data were collected through in-depth individual semi-structured interviews, which were conducted by researcher with extensive experience in qualitative studies and a PhD in nursing. The participants were the families of patients who had spent a minimum of three days in the ICU. A total of 11 participants were interviewed in this study. The time and place of the interviews were determined based on the participants’ preferences and a quiet space in the hospital training room was chosen for the interviews.

All the interviews, which lasted between 30 and 60 min, were conducted by the same researcher. The interviews started with the general question, asking participants to talk about their experiences in the ICU. Subsequent questions, such as “What problems did the ICU admission create for you?” “Would you please describe your experiences”? and “How did you feel?” were guided based on the information provided by the participant and the researcher’s desire to clarify relevant issues [15].

### Data analysis

The data were analyzed through the conventional content analysis method proposed by Graneheim and Lundman [17]. Firstly, each interview was transcribed verbatim by two researchers. Next, the interview transcripts were reviewed three times to obtain an overall understanding of the content. Then, each interview transcript was treated as the unit of analysis, and meaning units were identified and coded. The first author analyzed the total data, while the second one analyzed half of the textual data. The two authors compared their codes and revised minor disagreements through discussion. The codes were grouped into subcategories according to their conceptual similarities and differences. The subcategories were compared with each other, and the latent data content was identified and presented as categories. Finally, the categories were merged into three themes based on the latent data content. All authors reviewed the final nine categories and three themes to ensure a clear distinction between categories and subcategories and fit the data within each category.

### Trustworthiness

To ensure the accuracy of the data, four criteria were employed: credibility, confirmability, dependability, and transferability [16].

For credibility, participants with varying lengths of hospitalization and different diagnoses were included in the interviews. The participants were given the opportunity to review and confirm the codes extracted from the interviews and revise the contents, if necessary (member check).

To enhance confirmability, the interview texts, codes, and categories were evaluated by the research team (peer check) and two faculty members external to the research team (faculty check). The analyses were also presented to some participants, and their feedback was taken into consideration.

To ensure dependability, the article provided a clear presentation of the steps taken in data analysis, allowing other researchers to utilize the study’s data.

The participants were selected by maximum variation in terms of gender, age, relationship to the patient, and education, which enhanced the transferability of the study findings of the study.

### Ethical consideration

Ethical considerations were observed during the research process. Necessary permissions were obtained from the ethics committee of Kerman University of Medical Sciences (IR.KMU.REC.1402.185) on 25.09.2023. Written informed consent was obtained from the participants, ensuring that they could withdraw from the study at any stage without affecting the treatment process of their patients. The confidentiality of the information provided was emphasized, assuring participants that their identity would not be disclosed and the data would only be used for research purposes. Additionally, the method of accessing the research results was communicated to the participants.

## Findings

Eleven close family members participated in this study, consisting of five women and six men with a mean age of 47 ± 6.46 years. Among the participants, four had a diploma, six had a bachelor’s degree, and one held a master’s degree (Table 1). Table 1 presents the characteristics of participants (*n* = 11).

**Table 1:** Demographic information of study samples

Demographics		Participants	
		*N*	%
**Gender of the family**	Male	6	54.5
	Female	5	45.5
**Education** **of the family**	Diploma	4	36.4
	Bachelor’s Degree	6	54.5
	Master’s Degree	1	9.1
**Relationship to the patient**	Father	2	18.2
	Mother	4	36.4
	Spouse	3	27.2
	Siblings	1	9.1
	Child	1	9.1
	**Mean (SD)** 47(6.4)		
**Family age**			
**Age of the patient**	52.4(7.2) 21.7(6.2)		
**The length of stay in the ICU(Day)**			

Three themes emerged from the data analysis: the search for support sources, psychological consequences within the family, and the presence of various needs within the family. These themes provide valuable insights into the complex experiences of family members in ICU settings (Table 2).

### A: the search for support sources

Families of patients admitted to the ICU often face concerns and confusion. As a result, the search for support resources emerged as a coping strategy that can be further categorized into two subcategories: external and internal support resources.

### 1-External support sources

The support and encouragement from family, friends, and healthcare team members can enhance the sense of hope within families.

*“My husband has been hospitalized for 15 days, and my family has been my sole source of encouragement and support throughout this challenging period. Their presence has been crucial in helping me navigate the stress and difficulties associated with this situation.” (P2)*.

*“I find myself constantly waiting outside the ward, a familiar face to the nurses who have come to know me well. They often offer comforting words and understanding, allowing me to be by my loved one’s side.” (P5)*.

*“The expenses associated with treatment and medications are incredibly high. Without the support of my friends and family, I would not have been able to endure this financial burden for such a prolonged period.” (P4)*.

### 2-Internal support resources

Families also found solace in their religious practices, such as reading prayers and the Quran. These spiritual resources played a crucial role in alleviating stress and anxiety among the patients’ families.

*“I maintain a strong belief in my patient’s recovery. I have made a solemn vow to him, and in pursuit of this promise, I find solace in reading prayers and the Quran behind the closed doors of the ICU.” (P5)*.

### B: psychological consequences within the family

As time went on during the patient’s hospitalization, the families experienced various psychological consequences. They had concerns and fears about the unknown condition of the patient and the environment of the ICU. However, certain interactions with the staff could instill a sense of hope. Families, in their efforts to maintain hope, experienced feelings of rejection when they were excluded from decisions regarding the patient and their opinions and expectations were disregarded. These experiences can be categorized into four subcategories as follows:

### 1-Concern about the patient’s condition

The families of the patients reported experiencing significant stress regarding their loved one’s condition. Many of them were worried about the patient’s lack of consciousness or their inability to be present at the bedside. The unclear cause of the disease and its diagnosis further heightened their fears of losing their loved one.

*“I desire to enter the ward and witness the recovery of my patient. There is nothing I desire more than someone to lend an ear, listen to me, and alleviate my worries.” (P3)*.

*“I desperately crave reassurance that my patient will recover. I am flooded with questions about the duration of his mechanical ventilation, the cause of his stomach bleeding, and the necessity of the surgery he underwent. The constant fear haunts my thoughts, fearing that my patient may not survive.” (P5)*.

### 2-The frightening environment of the ICU

The fear and anxiety experienced by the patients’ families were often triggered by the unfamiliar and intimidating environment of the ward and the various medical devices connected to the patient. The unknown nature of the environment heightened their fear.

*“I felt extremely frightened when I saw my mother. The urge to cry overwhelmed me. Tubes were attached to her, and there were multiple serum samples. The weight of the connections on my mother were overwhelming.” (P8)*.

### 3- Hopeful interactions

During stressful situations, many healthcare team members, including nurses and doctors, demonstrated a deep understanding of the family’s situation. They provided reassurances about the patient’s condition, instilling hope in the midst of uncertainty.

*“Certain healthcare workers diligently addressed our inquiries about the patient, offering consolation and instilling hope by assuring us of our loved one’s improving condition.” (P5)*.

*“During the meeting with the doctor to discuss my father’s surgery, he thoroughly explained the procedure to us and reassured us that the surgery would lead to an improvement in my father’s condition. The doctor’s words had a calming effect on us, alleviating our concerns.” (P9)*.

### 4- Feelings of rejection

The families of the patients experienced increased concerns and stresses due to inappropriate treatment, inability to be at the patient’s bedside, and restrictions on visitation due to the patient’s deteriorating condition. These resulted in feelings of despair and separation between the family and the patient.

*“I am prohibited from entering the department. The nurse claims that my presence disrupts the patient’s vital signs. However, I have observed that when I shake their hand, the patient recognizes my presence.” (P1)*.

*“Whenever I spend time with my patient, I am promptly asked to leave the ward, with the explanation that prolonged presence may increase the risk of infection. However, I strongly believe that my presence has a positive impact on my patient’s well-being and can contribute to their recovery.” (P10)*.

### D: the presence of various needs within the family

During the patient’s admission to the ICU, the family expressed several needs arising from the prolonged stay. These needs shed light on the insufficient facilities available for these families. This category encompasses the need for comfort and convenience, the need for sympathetic assurance, and access to information sources.

### **1- the need for comfort and convenience**

The absence of a designated resting area, inadequate nutrition, insufficient sleep, and fatigue associated with prolonged hospital stays were factors that heightened the discomfort experienced by the patients’ families.

*“I have been in the hospital for several days, without a proper place to sleep or enough food to eat. However, I remain hopeful that this situation will improve. My greatest wish is for my patient to recover and for us to find happiness once again.” (P1)*.

*“I am physically and mentally exhausted, reaching a point where I feel unable to continue. The persistent lack of sleep has only intensified my fatigue.” (P6)*.

### **2- the need for sympathetic assurance**

The patients’ families expressed various needs, including support from families and friends, the desire to discuss the patient’s condition, reassurance from the medical staff regarding the patient’s recovery, addressing financial concerns, experiencing overwhelming sadness, and expressing emotions through crying.

*“I feel overwhelming sadness, and all I want to do is cry. During these difficult times, I yearn for someone to talk to and provide me with support. I feel incredibly lonely, as I have always carried my burdens alone. It feels as though I have no one to turn to.” (P11)*.

*“I long for someone to provide me with reassurance that my patient will recover. It would be immensely helpful if someone explain the patient’s condition to me in a clear and understandable manner.” (P4)*.

### **3- Access to information sources**

The patient’s family recognized the importance of receiving education and information during hospitalization. They expressed a need to be informed about various aspects, including the patient’s condition, ward environment, patient care procedures, diagnosis, and the underlying cause of the disease, attending doctor, equipment connected to the patient, and the discharge schedule and timing.

*“I hope for the opportunity to meet with my patient’s doctor and nurse, as it would greatly benefit us to have them explain the treatment process in a way that we can fully comprehend.” (P4)*.

*“I strongly desire to receive training regarding the care provided to my mother, as it would enable me to take care of her independently upon her discharge.” (P8)*.

**Table 2:** Themes, major categories, and subcategories extracted from the data

Theme	categories	Subcategories	primary code
Search for support sources	External support sources	Hopeful support of relatives	Good support from the wife
			Continuous access to family members and relatives
			Support of children
			Support from close friends
			Emotional support of relatives
			Continuous disobedience of relatives of the patient
			Cooperation and empathy of all family members during hospitalization
		Financial support of relatives	Procurement of rare drugs by relatives
			Financial support from relatives
			Donations from relatives
			Preparation of necessary equipment for the patient by all family members
		Healthcare team’s support	Psychological support of nurses
			The concern and empathy of the department staff for the family
			The kind behavior of the ward personnel with the family
			Encouraging words of the doctor
	Internal support sources	Sources of beliefs	Surrender to the will of God
			Assurance of God’s help
			Acceptance of a divine exam
		Religious sources	Giving offerings to imams
			Connecting things, bless the patient
			Giving blessed food
		Religious rituals	Pray
			Reading Quran
			Prayers for the sick
			Tawassul
Psychological consequences within the family	Concern about the patient’s condition	Fear of separation from patients	Concern about not having the opportunity to see the patient
			Anxiety due to not being able to be beside the patient
			Fear of being alone in a critical situation
		The lack of awareness of the patient’s condition	Ignorance of the possible complications of the procedures
			Absence during the visitation hours
			Uncertainty about the time of patient visits
			Unfamiliarity with the devices connected to the patient
			Lack of awareness of test results
	The frightening environment of the ICUs	The fear of an unfamiliar environment	Fear of the conditions of other hospitalized patients
			Unfamiliarity with the department environment
			Concern about the large number of personnel in the department
			Concern about inadequate usage of infection control equipment while attending to the patient
			Fear of the confined environment
		The fear of unfamiliar devices	Fear of multiple patient connections
			Feeling anxious when encountering unfamiliar equipment in the ward
			Fear of the noise generated by medical devices
	Hopeful interactions	Effective communication with the healthcare team	Nurses’ interaction with the family regarding the patient’s recovery
			Considering the family’s emotional state
			Explaining the patient’s condition to alleviate worry
		Patient exposure to the treatment team	Instilling a sense of hope in the patient’s companions by the nurse
			Listening patiently to the nurse
			Listening to the concerns of the doctor
			Showing empathy towards the family during the critical condition of the patient
	Feelings of rejection	Ignoring families’ expectations	Inadequate explanation to the patient’s family
			Limited consideration given to the opinions of the family
			Lack of communication regarding the family’s numerous questions
			Family concerns left unanswered by the staff
		Non-participation of the family in decision-making	Making decisions about interventions without involving the family
			Family members being unaware of patient transfer plans
The presence of needs within the family	The need for comfort and convenience	Physical facilities for the families	Need for a private space
			Availability of prayer facilities in the waiting room
			Need for a phone near the waiting room
			Insufficient seating arrangements in the ward corridor
		Physiological needs of the families	Lack of access to proper meals
			Not getting enough sleep
			Not getting enough rest
			Absence of basic physiological requirements
			Lack of access to toilets near the ward
	Seeking sympathetic assurance	Need to be seen and talk	Desire for interaction with other companions
			Need for communication with the doctor
			Need for communication with the nurse
		Effective assurance	Ensuring the importance of staff attentiveness to the patient
			Ensuring optimal patient care
			Ensuring a good patient outcome
	Access to information sources	Awareness of the patient’s condition	Awareness of discharge time
			Understanding when to stop ventilator support
			Knowledge of the cause of the disease
		Training during discharge	Post-discharge care guidelines
			Knowledge of referrals after discharge
			Guidance on home-based patient feeding
			Instructions for patient care during the discharge process

## Discussion

This study analyzed the experiences of families with loved ones admitted to the ICU, resulting in the identification of four themes. The study findings indicated that families actively sought various sources of support to cope with the immense stress and adapt to their circumstances. External support from families, friends, and the healthcare team was a significant source of encouragement during this challenging period. Additionally, families found solace and ease in their internal support systems such as their religious beliefs, spiritual values, and practices.

The study findings align with the research conducted by McKiernan et al. (2010), who also emphasized the crucial role of the healthcare team in supporting families. Being in close proximity to both patients and families, the healthcare team was able to address their concerns, answer their questions, and offer assistance during this difficult time [18]. Similarly, Gaeeni et al. (2018) highlighted the significance of support from families, friends, the healthcare team, as well as religious resources and beliefs in managing and adapting to the stress caused by the hospitalization of a loved one.

The experience of hospitalization imposes significant demands and challenges on families, leading to changes in their daily lives. However, utilizing support resources can help meet these demands and stabilize family functioning [19]. Digby et al. (2023) found that effective communication with healthcare staff was highly important to the participants. The quality of communication varied and depended on the staff behaviors. Participants generally perceived communication with nurses as more effective than with doctors, which instilled confidence in them [20].

Religious beliefs, particularly among Muslims, have a significant impact on reducing stress and anxiety. Muslims often turn to God through prayer and supplication when facing critical situations, seeking his help [13].

Another challenge faced by families was the psychological consequences. The intimidating environment of the ICU, the presence of various medical devices connected to the patient, alarms of these devices, presence of critically ill patients in the ward, and occurrence of patient deaths in these units, coupled with restricted access to the patient’s bedside, all contribute to the fear and anxiety among families. Additionally, the lack of information about the patient’s physical condition constantly concerned the families, fearing deterioration and potential loss.

Several studies have provided evidence that close family members of ICU patients are susceptible to experiencing anxiety, depression, post-traumatic stress, and complex grief [21–24]. The recovery process for patients’ families following the ICU hospitalization often takes longer than that of the patients themselves [25]. The separation of families from their loved ones in the ICU also had significant negative effects. Being separated from their ill relatives causes immense distress for families. This separation, coupled with limited opportunities to establish a relationship with the healthcare staff, leaves some families feeling disconnected and anxious [13].

Families find themselves caught in a whirlwind of uncertainty, shock, despair, anxiety, depression, helplessness, and confusion due to the fear of their loved one’s death, uncertainties surrounding the prognosis and treatment, emotional disturbances, concerns about the financial burden associated with the patient’s hospitalization, role changes, and disruptions in daily life [26].

Families of patients admitted to the ICU reported both hopeful interactions and feelings of rejection. Hopeful interactions included receiving empathy and understanding from the healthcare team, providing adequate information, being treated with respect, and receiving hope and reassurance about the patient’s recovery. On the other hand, feeling rejected involved actions such as separating the family from the patient, disregarding their needs, treating them inappropriately, and excluding them decision-making.

Various studies have demonstrated that family members of ICU patients face a high risk of psychological burden. Two main risk factors for families are an increased psychological burden and poor communication with the ICU team. Effective communication with the ICU team, both during the patient’s stay and after their passing, plays a crucial role in supporting and comforting family members [25–28]. Inadequate, unsatisfactory, or distressing communication between the healthcare team and families has been associated with a higher risk of stress for the families (29–30). When relatives perceive the information provided as incomplete, the risk of experiencing symptoms related to post-traumatic stress disorder (PTSD) increases [31].

Another study found that visitation restrictions imposed on close family members of ICU patients caused significant distress for some families. Accepting the limitations on visitation and experiencing separation was extremely challenging for certain participants, leading to feelings of anger and frustration [13].

Effective communication between nurses and patients’ families is a crucial aspect of providing quality care and emotional support to the families [32]. Furthermore, to understand the emotional impact of a patient’s ICU hospitalization and meet the needs of the patient’s family, nurses must establish effective interactions with the families [33]. However, Biancofiore (2010) highlighted a negative belief among nurses regarding family involvement, as they may perceive themselves solely responsible for patient care and overlook the family needs [34]. Therefore, one of the main reasons for visitation restrictions is the presence of negative beliefs and a lack of belief in the benefits of family visits for the patient [35].

During the hospitalization of their loved ones, families faced various challenges and had numerous needs. They expressed a desire for comfort and convenience. Issues such as nutrition, adequate rest and sleep, clarity about the happenings behind the doors of the ICUs, companionship, and effective communication were not adequately addressed. Additionally, they experienced uncertainty regarding the patient’s condition, a lack of effective training provided to the family, feelings of abandonment, and a lack of family-centered care.

Another finding of this study was the importance of access to information sources. Studies have shown that families of patients admitted to ICUs have diverse needs, including physical, emotional, psychological, and informational needs. Obtaining information and education was identified as one of the most crucial needs for families (36–37). Another significant need for families is to be close to the patient and have information about their condition. Meeting this need can greatly fulfill the psychological and emotional needs of the family [38]. Furthermore, a study revealed that family members required honest information about the patient’s progress to help them cope with the situation. They emphasized the importance of “situation awareness,” which involved continuously monitoring and evaluating the care provided to the patient [18]. Being able to be with their relatives helped maintain the family bond and served as a way to demonstrate love and support for the patient. The reassurance of care provided by nurses also created a sense of security for the patients’ families.

## Conclusion

The study findings revealed that when families were present in the ICU, they actively sought support resources due to their fear of losing the patient. Interactions with the healthcare team and the fulfillment of their needs could significantly affect their sense of hope and confidence in the patient’s condition. It is crucial to recognize the importance of providing support and attention not only to the patients but also to their families, based on the experiences shared by the participants. Therefore, it is recommended that managers, doctors, and other healthcare members prioritize and actively support the families in order to effectively contribute to the patient’s overall well-being. Furthermore, the knowledge gained from this study can assist healthcare professionals, particularly doctors and nurses, in implementing family-centered approaches and interventions.

## Data Availability

The datasets used during the current study are available from the corresponding author on reasonable request.
